# Cranberry-Derived Phenolic Compounds Contribute to the Inhibition of FimH-Mediated *Escherichia coli* Hemagglutination

**DOI:** 10.3390/antibiotics14040418

**Published:** 2025-04-21

**Authors:** Rosana Ribić, Vesna Petrović Peroković, Tomislav Meštrović, Marijana Neuberg, Nikola Bradić

**Affiliations:** 1 University Centre Varaždin, University North, HR-42000 Varaždin, Croatia; rribic@unin.hr (R.R.); mneuberg@unin.hr (M.N.); nbradic@unin.hr (N.B.); 2 Department of Chemistry, Faculty of Science, University of Zagreb, HR-10000 Zagreb, Croatia; vpetrovi@chem.pmf.hr; 3 Department of Anesthesiology and Intensive Care, University Hospital Dubrava, HR-10000 Zagreb, Croatia

**Keywords:** *Escherichia coli*, hemagglutination inhibition assay, FimH adhesin, mannose, cranberry-related phenols, urinary tract infection

## Abstract

**Background/Objectives:** FimH adhesin, located at the tips of type 1 pili in *Escherichia coli* (*E. coli*), plays a crucial role in bacterial adhesion to the surface urothelial cells—a key step in the pathogenesis of urinary tract infections (UTIs). Given the rising concern over antimicrobial resistance (AMR), and considering that *E. coli* is one of the pathogens with the largest AMR burdens on a global scale, alternative strategies targeting bacterial adhesion are gaining increasing attention. Products that contain D-mannose and cranberry-derived phenolic compounds have shown promise in preventing *E. coli* colonization and infection. The aim of this study was to investigate the antiadhesive effects of cranberry-related phenolic compounds on FimH-mediated *E. coli* adhesion using a cellular hemagglutination inhibition assay, as well as to assess the synergistic effects of mannose and phenolic compounds on biofilm formation. **Methods:** A range of phenolic acids (benzoic, chlorogenic, hippuric, *p*-coumaric, ferulic and caffeic), resveratrol, (+)-catechin and procyanidin A, as well as a *Vaccinium macrocarpon* extract, were evaluated for their ability to inhibit FimH-mediated adhesion. A binocular microscope was used to observe agglutination, and we also evaluated the biofilm inhibition potential of the phenolic compounds in the presence of D-mannose. **Results:** Our results demonstrated that these compounds significantly reduced hemagglutination, with benzoic acid, chlorogenic acid, caffeic acid and resveratrol exhibiting strong inhibitory effects at concentrations as low as 0.25 mM. Furthermore, the addition of 1 mM solutions of these phenolic compounds to D-mannose resulted in a twofold reduction in the inhibition titer, suggesting synergistic interactions. In addition to their antiadhesive properties, the tested phenolic compounds contributed slightly to the inhibition of FimH-mediated biofilm formation, further supporting their potential roles in UTI prevention. **Conclusions:** These findings highlight the potential of cranberry-derived phenolics as natural antiadhesive agents against *E. coli* and warrant further investigation into their mechanisms of action and possible applications in infection control.

## 1. Introduction

Infectious diseases remain one of the leading causes of death and a global public health challenge. Urinary tract infections (UTIs) are among the most common bacterial infections, particularly affecting females, and they are predominantly caused by uropathogenic *Escherichia coli* (UPEC) [1]. UTIs pose a significant health challenge due to their high recurrence rates; moreover, the frequent use of antibiotics as the first-line treatment contributes to the growing issue of antimicrobial resistance (AMR), with *E. coli* being one of the principal AMR burden generators [2]. The resistance of UPEC to commonly prescribed antimicrobial agents has risen, leading to increased treatment costs and resistance to multiple antibiotics, with substantial implications for the healthcare systems. In addition, persistent UTIs can cause pyelonephritis, leading to the activation of inflammatory mediators and the overproduction of reactive oxygen species [3]. Therefore, the need for new strategies for the prevention and treatment of UTI has emerged, with one of the most promising being antiadhesion therapy [4]. Namely, the first step in UTI pathogenesis is the adherence of specific bacterial surface-associated proteins, bacterial adhesins, to complementary antigens of the eukaryotic cell. Adhesive organelles, which extend from the bacterial surface, are called pili or fimbriae. Common adhesive organelles in UPEC are fimbriae type I, P, S and F1C pili. Pivotal virulence factors and, therefore, the most studied ones are type I fimbriae and P pili [4].

FimH adhesin, located at the tops of type I fimbriae, specifically recognizes mannosylated glycoprotein uroplakin 1a on the surfaces of urothelial cells, enabling bacterial invasion, colonization, proliferation and subsequent biofilm formation. FimH blocking with antagonists represents as a promising new therapeutic approach for the treatment of infections caused by UPEC. The natural ligand D-mannose binds to FimH with modest affinity; however, its affinity can be significantly improved via the modification of mannose at the C-1 position by introducing various lipophilic (alkyl and aryl) α-mannosidic aglycones [5]. In general, mannose moieties from FimH antagonists establish hydrogen bond networks within the carbohydrate recognition domain, and a lipophilic aglycone interacts with the hydrophobic amino acids placed at the entrance to the carbohydrate recognition domain [6]. Clinical studies have shown that D-mannose powder can significantly reduce the risk of recurrent UTIs [7,8].

Beyond UTIs, FimH antagonists have potential for the treatment of Crohn’s disease (CD). Adherent invasive *E. coli* (AIEC) is particularly present in the ileal mucosae of patients suffering from CD, and there is significant overlap between the AIEC and UPEC pathotypes, suggesting that factors that enhance virulence in the urinary tract may also enhance pathogenicity in CD patients [5]. AIEC binds to ileal enterocytes. Adhesion is supported by the excessive expression of carcinoembryonic antigen-related cell adhesion molecule 6 (CEACAM6), and thus an increased number of mannoses expressed on the apical sides of epithelial cells of the ileal mucosa allows the stronger binding of AIEC [9].

P-type fimbriae were the first characterized virulence factors of UPEC, and they are responsible for mannose-resistant adhesion. Adhesion protein PapG is placed at the tops of the P pili and binds to digalactoside (galabiose) units found on the surface epithelial cells lining the urinary tract [10]. Akin to the approach used for the design of FimH antagonists, synthetic galactosides with aromatic substituents have been developed. Due to exhibited binding affinity toward PapG, they can inhibit PapG-mediated adhesion [6].

The use of cranberry extracts is widely recommended for patients with recurrent UTIs. It has been proposed that cranberry extracts inhibit UPEC adhesion. A-type proanthocyanidins (PACs) have been suggested as being mainly responsible for the prevention of UTIs by blocking P-fimbriae [9,11,12]. On the other hand, in a recent study, PAC-free extracts also showed significant antiadhesive effects [13,14]. Therefore, the literature data indicate that there are open questions related to active cranberry ingredients, especially phenolic compounds, and their corresponding biological activity that remain unanswered. The cranberry is rich in several groups of phenolic compounds, simple phenols and phenolic acids (benzoic, phenylacetic, phenylpropionic and cinnamic acids), flavonols, anthocyanins and PACs [15]. Other phytochemicals that occur in cranberries (besides polyphenols) are terpenes, organic acids and carbohydrates [16]. Polyphenols display strong antioxidant and anti-inflammatory properties and an ability to facilitate some chronic diseases and slow down the inflammatory process [17]. A positive correlation has been found between the number of hydroxyl groups and antioxidant activity, as well as the synergistic, additive and antagonistic effects of antioxidants [3].

In general, lectins FimH and PapG, found on these adhesive organelles, contain high levels of hydrophobic amino acid residues and therefore cause the bacterial cell surface to exhibit hydrophobicity, which is conducive to adhesion to host cells [18]. Aromatic residues have been implicated in binding carbohydrates [19]. The dual target binding of mannose and phenolic moieties could block the infection and prevent disease progression more efficiently. Therefore, it represents a potential foundation for further research [20]. In this study, we aimed to investigate the influence of phenolic compounds on FimH-mediated *E. coli* adhesion. The contribution of phenolic compounds derived from cranberry extracts to the D-mannose-induced inhibition of *E. coli* adhesion was assessed using the hemagglutination inhibition assay. We also aimed to assess the synergistic effects of mannose and phenolic compounds on biofilm formation.

## 2. Results

The influence of natural phenolic compounds related to cranberries on FimH-mediated *E. coli* hemagglutination was evaluated using HAI assays and expressed in ITs. The IT denotes the minimal concentration of the tested compound required to prevent type 1 fimbriated *E. coli* HB101(pPKL4) from agglutinating guinea pig red blood cells. Phenolic compounds were screened in order to determine their potential antiadhesive properties towards type 1 fimbriated *E. coli* HB101(pPKL4); these specifically recognized mannosylated structures. Therefore, mannose was used as reference compound, while sixteen phenolic compounds and an extract of *Vaccinium macrocarpon* were biologically evaluated. The following phenolic compounds were tested: catechol, benzoic acid, diphenylacetic acid, *p*-coumaric acid, ferulic acid, caffeic acid, chlorogenic acid, quercetin, catechin, epicatechin, resveratrol, curcumin and procyanidin A2. The standard protocol for HAI was used, in which a serially diluted solution of the tested pure compounds or a mixture of mannose with phenolic compounds was placed into a 96-well V-shaped microtiter plate and then mixed with suspensions of red blood cells and bacteria with a defined concentration. After incubation, the difference between the agglutination (diffuse reddish mats) and sedimentation of non-agglutinated erythrocytes (red dots) was interpreted. The results of the HAI assay were also confirmed by microscopic detection. The obtained results were reproducible, and the values and derived binding capacities were identical for all independent tests performed.

The results of the first HAI assay, in which solutions containing a mixture of mannose in serially diluted concentrations and 1 mM solutions of phenolic compounds were tested, are shown in Table 1. In this assay, D-mannose (Man) served as the reference compound.

The inhibition titer (IT) values for the mixtures of Man and catechol, diphenylacetic acid, curcumin, quercetin and epicatechin were 60 mM, the same as for the pure Man. When solutions containing Man and benzoic acid, hippuric acid, *p*-coumaric acid, ferulic acid chlorogenic acid, resveratrol, catechin, procyanidin A2 and extracts of *Vaccinium macrocarpon* (*V. macrocarpon*) were used, IT values of 30 mM were obtained. Based on the described HAI assay, in which most promising phenolic compounds were determined, an HAI assay was performed with the use of serially diluted solutions of selected phenolic compounds (starting from a 1 mM concentration). The results of this HAI assay are shown in Table 2.

**Figure 1:**
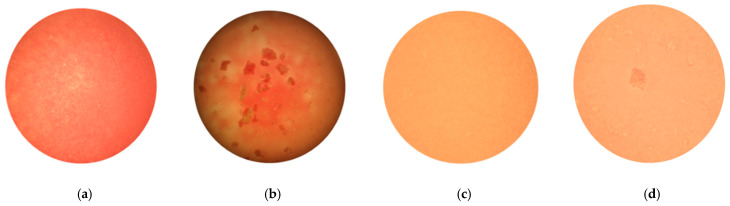
Representative images of microscopic inspection of HAI of (**a**) a 5% suspension of guinea pig erythrocytes; (**b**) a mixture of erythrocytes and type 1 fimbriated *E. coli* HB101(pPKL4), resulting in hemagglutination; (**c**) a mixture of erythrocytes and type 1 fimbriated *E. coli* HB101(pPKL4) in the presence of a 60 mM mannose solution, where the inhibition of hemagglutination occurred; (**d**) a mixture of erythrocytes and type 1 fimbriated *E. coli* HB101(pPKL4) in the presence of a 30 mM mannose solution (without the addition of phenolic compounds), where the inhibition of hemagglutination did not occur.

All selected compounds, including the *V. macrocarpon* extract, inhibited hemagglutination at lower concentrations than in the previous HAI screening. The lowest determined IT values were 0.25 mM, and they were obtained for benzoic acid, caffeic acid, chlorogenic acid and resveratrol, while the IT for the *V. macrocarpon* extract was 10 mg/mL. The inhibition of hemagglutination in the presence of mannose and/or in the presence of the tested phenolic compounds was also examined by microscopic detection. Microscopic detection confirmed the described results of the HAI assays. Representative images are shown in Figure 1.

**Figure 2:**
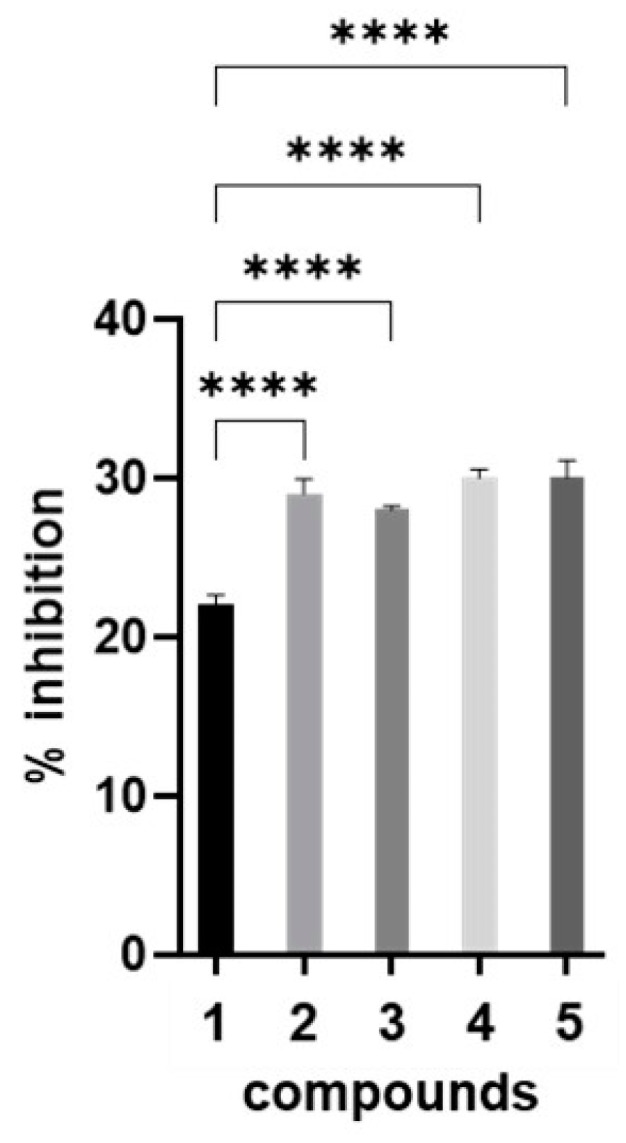
Inhibition of biofilm formation of type 1 fimbriated *E. coli* HB101(pPKL4) by a 30 mM solution of mannose (1) and mannose (30 mM) mixed with 0.25 mM phenolic compounds, i.e., benzoic acid (2), chlorogenic acid (3), resveratrol (4) or caffeic acid (5). **** *p* < 0.0001 denotes statistical significance between indicated groups.

The most potent phenolic compounds (benzoic acid, caffeic acid, chlorogenic acid and resveratrol) in the presence of 30 mM mannose were then further evaluated using a biofilm inhibition assay. The bacterial suspension used in the previously described HAI assay was incubated for 24 h with a mixture containing mannose and these four phenolic compounds at a concentration of 0.25 mM. Biofilm inhibition was analyzed using crystal violet staining and absorbance measurements at 590 nm. The results, shown in Figure 2, indicated that the tested phenolic compounds contributed slightly to the inhibition of FimH-mediated biofilm formation.

In the biological evaluation of novel compounds with potential dual or synergistic activity, testing non-conjugated compounds is a crucial initial step. This approach provides valuable insights that guide subsequent chemical modifications and optimization strategies. The experiments described here exemplify this type of initial screening, which allows researchers to identify the most promising compounds without the added complexity of conjugation.

## 3. Discussion

Since the consumption of cranberry (*V. macrocarpon*) and mannose is recommended for prophylaxis against UTIs [21], the aim of this study was to investigate the antiadhesive properties of cranberry-related phenolic compounds towards FimH-mediated *E. coli*, which is known to be mannose-specific. The influence of natural phenolic compounds on *E. coli* adhesion was studied using HAI, and the antiadhesive potential of the tested samples was expressed in ITs. In order to examine the influence of phenolic compounds specifically on FimH-mediated adhesion, FimH adhesins were expressed in *E. coli* HB101, an avirulent *E. coli* strain. It is known that the expression of type 1 fimbriae enhances *E. coli*’s virulence for the urinary tract [22,23].

Recent meta-analyses reinforce the value of non-antibiotic interventions in preventing UTIs in both children and adults, highlighting alternatives to long-term antibiotic use (and antiadhesive approaches akin to ours belong to this group). For example, Meena et al. demonstrated that cranberry products are as effective as antibiotic prophylaxis (relative risk [RR]: 0.92, 95% confidence interval [CI]: 0.56–1.50) and significantly better in reducing UTI recurrence when compared to a placebo (RR: 0.52, 95% CI: 0.29–0.94) [24]. Han and colleagues recently analyzed 50 randomized controlled trials with over 10,000 participants, identifying D-mannose and cranberry as effective preventive measures [21]. Notably, D-mannose exhibited the strongest reduction in the UTI incidence (RR: 0.34, 95% CI: 0.21–0.56). Importantly, these interventions did not result in a higher incidence of adverse effects compared to the placebo, reinforcing their safety. All of this means that there is a growing shift toward non-antibiotic UTI prevention strategies, which could in turn reduce antibiotic resistance while providing effective long-term management solutions.

The HAI assay, commonly used to evaluate FimH-mediated bacterial adhesion, involves the bacterial lectin FimH as the receptor and the erythrocyte surface glycocalyx as its natural, highly effective ligand [21]. For screening, the classical cellular inhibition hemagglutination assay was used, rather than a cell-free FimH binding assay [23], since it directly estimates the compound’s ability to prevent bacterially mediated adhesion, where the tested antagonist competes with erythrocytes (as demonstrated in other studies) [25,26]. In this study, a *V. macrocarpon* extract and phenolic compounds—simple phenols such as catechol; aromatic acids (benzoic, diphenylacetic and several cinnamic acids, namely *p*-coumaric, ferulic, caffeic and chlorogenic); flavonoids (quercetin, catechin, epicatechin); a stilbenoid, namely resveratrol; curcumin; and procyanidin A2—were included. The IT values for the mixtures of Man with catechol, diphenylacetic acid, curcumin, quercetin and epicatechin were 60 mM, the same as for pure Man. It is known that Man has a twofold higher IT than methyl-α-D-mannopyranoside (MeMan), which is often used as a reference compound [27]. Meanwhile, the ITs for the mixtures of Man with aromatic acids—benzoic, hippuric, *p*-coumaric, ferulic and chlorogenic—and resveratrol, catechin, procyanidin A2 and extracts of *Vaccinium macrocarpon* were two times lower (30 mM).

The data obtained indicate that these phenolic compounds influence FimH-mediated *E. coli* adhesion, and they are in accordance with previously published results [28,29,30]. Namely, de Llano and colleagues showed that catechol, benzoic acid, vanillic acid, phenylacetic acid and 3,4-dihydroxyphenylacetic acid exhibit antiadhesive activity against UPEC (ATCC^®^53503™) in a concentration range of 100–500 μM and that procyanidin A2 inhibits agglutination at 500 μM [30]. They also confirmed the antiadhesive activity of cranberry procyanidins and their microbial-derived metabolites against *E.coli* ATCC 53503 and DSM 10791. A-type procyanidins (A2 and cinnamtannin B-1), and hippuric acid as a metabolite, exhibited antiadhesive activity at concentrations higher than 250 μM. Additionally, a methylated form of 3,4-dihydroxyphenylacetic acid (4-hydroxy-3-methoxyphenylacetic acid) was found to be less active than the corresponding 3,4-dihydroxyphenylacetic acid [31]. In our model system, where type 1 fimbriated *E. coli* HB101(pPKL4) was used, the IT for caffeic and chlorogenic acid was 0.25 mM. These results confirm the importance of the 3,4-dihydroxyphenyl motif in antiadhesive activity and are in good agreement with previous findings. Namely, ferulic acid, which is a 3-methoxy derivative of caffeic acid (3-(3,4-dihydroxyphenyl)-2-propenoic acid), shows a two times higher IT than caffeic acid. The biofilm inhibition assay also showed that the presence of two OH groups in a 3,4-relation to benzoic acid’s carboxylic group resulted in reduced biofilm formation by *E. coli.* These results are in good agreement with previously published data showing the influence of the positional isomerism of benzoic acid derivatives on antibacterial activity against UPEC [32]. The redox potential of the corresponding phenolic acids also increases in the order caffeic acid > ferulic acid > benzoic acid, showing a positive correlation between the number of hydroxyl groups and the redox potential [33]. Chlorogenic acid, the ester of caffeic acid and (−)-quinic acid, exhibits antiadhesive, antioxidant and anti-inflammatory properties in vivo and in vitro [34]. As a naturally occurring stilbene, resveratrol, which also possesses a 3,4-dihydroxyphenyl motif, as well as caffeic and chlorogenic acids, with antiadhesive activity, exhibit anti-inflammatory effects in intestinal cells [35]. Resveratrol inhibits several key components of the inflammation cascade, most commonly described by the inhibition of NF-κB activation [36].

In general, we can conclude that simple cranberry-related phenolic compounds, particularly benzoic acid, caffeic acid, chlorogenic acid and resveratrol, contribute to the inhibition of the adhesion of FimH-mediated *E. coli*. Although the biological effects of cranberry extracts could not be considered as the cumulative effect of separate compounds present in the cranberry composition or its metabolites, the contributions of certain phenolic compounds to the inhibition of FimH-mediated adhesion can be observed. Interestingly, the synergistic effect of phenolic compounds (catechin, protocatechuic and vanillic acids) was shown to be effective against the adhesion of UPEC on silicone catheters [37]. According to the literature, mannose—most notably, its derivatives with attached aromatic aglycones—has a more significant impact on FimH-mediated adhesion [4]. Additionally, the daily oral consumption of mannose proved to be effective and safe in preventing recurrent UTIs in women, and it was shown to be superior to the oral consumption of isolated PACs [38,39].

The obtained results could contribute to the development of more effective naturally inspired antiadhesives. In the context of FimH-mediated adhesion, the bioinspired mannosides containing phenolic aglycons show significant potential. Therefore, it is important to further investigate the biological activity of mannose conjugates with the most active phenolic compounds, especially caffeic acid, chlorogenic acid and resveratrol, which share the 3,4-dihydroxyphenyl motif. This study further supports the potential of natural phenolic compounds to enhance the antiadhesion effects, similarly to the phenols in propolis, which have been shown to improve antibiotics’ efficacy and reduce antibiotic resistance in UPEC. By exploring synergistic interactions, this research is expected to contribute to the development of novel strategies for the treatment of UTIs while concurrently mitigating the emergence of antibiotic resistance [40].

Furthermore, Trombino et al. have described multifunctional microspheres composed of D-mannose and resveratrol for the controlled release of ciprofloxacin. The incorporation of D-mannose enhances the delivery and efficacy of ciprofloxacin by promoting its interaction with bacterial surfaces, while resveratrol contributes to antioxidant properties, which may help to mitigate infection-associated oxidative stress; additionally, resveratrol may synergize with ciprofloxacin, further enhancing its antibacterial activity [41]. This represents one good example of a synergistic therapeutic system where the combination of mannose, a phenol compound with a 3,4-dihydroxyphenyl motif, and an antibiotic could provide enhanced antimicrobial effects. To the best of our knowledge, other conjugates of mannose and phenols with a 3,4-dihydroxyphenyl motif have not been described in the literature thus far. This absence of existing research underscores the potential for future studies, providing opportunities to discover new therapeutic applications and uncover the biochemical properties of these conjugates.

This is reinforced by recent studies that demonstrate a growing interest in targeting FimH-mediated adhesion as a therapeutic strategy against *E. coli*-associated infections, particularly UTIs. Nasi et al. identified aggregation-prone regions in FimH and developed peptide analogs that disrupt its folding or block its mannose-binding pocket, offering a novel antimicrobial strategy [42]. Zhou and coauthors addressed the complexity of FimH antagonist synthesis, introducing a nickel-catalyzed method that streamlines production and enhances yields, facilitating the development of potent inhibitors for UTIs and Crohn’s disease [43]. Moreover, Faustino et al. explored mannose-rich oligosaccharides as a natural alternative to antibiotic prophylaxis, demonstrating their efficacy in blocking UPEC adhesion and reducing inflammation [44]. These findings highlight diverse approaches to inhibiting FimH, reinforcing its role as a promising target for future therapeutic development.

Additionally, Mousavifar and colleagues recently focused on the development of potent FimH antagonists, which led them to the synthesis of a new series of C-linked glycomimetic FimH inhibitors (incorporating hydrophobic aglycones) designed to enhance binding interactions with the FimH “tyrosine gate” [45]. These antagonists exhibited sub-nanomolar affinity and significantly improved adhesion inhibition compared to previous analogs. Structural analysis through crystallography and molecular dynamics simulations confirmed their effective binding to FimH, with several key interactions that contributed to their high affinity and with no cytotoxic effects and no interference with antibiotic activity. Their findings highlight the potential of these glycomimetic antagonists as promising candidates for antiadhesive therapy, and further investigation into the synergistic effects between these and phenolic compounds (as demonstrated in our study) could pave the way for optimized antiadhesive strategies.

The mechanism underlying the observed synergistic effects could be explained by the conformational change of FimH, leading it to a state of higher mannose affinity. Namely, the combined use of mannosides and phenolics results in stronger adhesion, probably due to the bidirectional disruption of the FimH-mediated adhesion mechanism, i.e., direct competition by mannose in the binding pocket and allosteric modulation by phenolics [31]. This hypothesis should be further explored in mutational studies, especially molecular simulations, which could describe the dynamics in the inhibitor binding process [46].

## 4. Materials and Methods

### 4.1. Materials

Mannose, sixteen phenol reagents and ascorbic acid were obtained from the Merck Corporation (Darmstadt, Germany), while chemicals for the preparation of the buffer were obtained from Kemika (Zagreb, Croatia) and the extract of *V. macrocarpon* (80 mg/mL) from Fidifarm (Rakitje, Croatia). Mannose was dissolved in phosphate-buffered saline (PBS) (pH 7.0), giving a stock solution with a 240 mM concentration. Pure phenolic compounds were weighed individually and dissolved in dimethyl sulfoxide (DMSO). Prepared DMSO solutions of the phenolic compounds were diluted in PBS to achieve stock solutions with a 1 mM concentration. All tested phenolic solutions contained a maximum of 0.03% DMSO (DMSO did not affect cells compared to untreated control cells).

Where applicable (i.e., the biofilm inhibition assay), the resulting data were analyzed using one-way ANOVA followed by Tukey’s post hoc test to assess the statistical significance between compounds. Results are reported as mean values ± standard deviations (SD), and differences were considered statistically significant at *p* < 0.05. The statistical analysis was conducted in the GraphPad Prism software version 10.1.2 (GraphPad Software, Boston, MA, USA).

### 4.2. Cultivation of Bacteria

The pPKL4 plasmid, bearing genes for *E. coli* type 1 fimbriae [47], was electroporated into *E. coli* HB101 strain-competent cells, followed by the selection of the transformed cells [HB101(pPKL4)] on LB/amp plates. A single colony was transferred into 500 mL of LB/amp media, and the bacterial culture was grown overnight at 37 °C (about 15 h). *E. coli* HB101(pPKL4) cells were harvested by centrifugation (450× *g*, 15 min at 4 °C), washed twice with PBS buffer (8 g NaCl, 0.2 g KCl, 1.44 g Na_2_HPO_4_ × 2H_2_O and 0.2 g KH_2_PO_4_ were dissolved in distilled water up to a 1 L total volume, pH 7.0) and resuspended in the same buffer in an approximate concentration of 10 mg/mL wet weight [20,40]. The produced *E. coli* HB101(pPKL4) cells were kept at 4 °C prior to their usage in the assay. The mentioned pPKL4 plasmid was generously gifted by Dr. Per Klemm, Department of Systems Biology, Technical University of Denmark. The plasmid was electroporated into *E. coli* HB101 strain-competent cells (defective in type 1 fimbriae production), followed by the selection of the transformed cells (HB101 [pPKL4]) on LB/amp plates.

### 4.3. Blood

Fresh guinea pig blood (5 mL), obtained from the Institute of Immunology, Department for Research and Development (Zagreb, Croatia), was stabilized by 3.8% sodium citrate (1 mL) and then centrifugated (450× g, 10 min), giving a sediment of packed erythrocytes (4 mL). The erythrocytes were carefully suspended in PBS (4 mL). The centrifugation and washing of the erythrocyte sediment were repeated twice. The final 5% suspension of guinea pig erythrocytes was obtained via the suspension of packed erythrocytes (4 mL) in PBS (76 mL). The suspension was stored at 4 °C prior to its usage in the assay.

### 4.4. Hemagglutination Inhibition (HAI) Assay

As already mentioned, all tested compounds were dissolved in PBS. First, in V-shaped 96-well microtiter plates (Nunc), 20 µL serially diluted mannose solutions (240 mM stock solution was twofold diluted, giving solutions with 120 mM, 60 mM, 30 mM, 15 mM, 7.5 mM, 3.75 mM, 1.88 mM concentrations) or 10 µL of serially diluted mannose solutions with 10 µL of the tested phenolic solutions were mixed with 20 µL of the bacterial suspension; afterwards, a 5% suspension of guinea pig erythrocytes (20 µL) was added. The plate was incubated at room temperature for 10 min and the assay was visually inspected, as in our previous study [48]. The lowest mannose concentration that inhibited hemagglutination was determined as the inhibition titer (IT). ITs were obtained from two independent tests where serially twofold diluted solutions were used in duplicate. In the second screening, the most potent phenolic compounds were evaluated by an HAI assay in which serially twofold diluted solutions of the phenolic compounds were used, starting from a 1 mM concentration.

### 4.5. Microscopy Examination of HAI

Microscopic detection was conducted using a modified procedure previously described in [49]. A total of 20 µL of the erythrocyte suspension with a solution of a defined concentration of the tested compound was thoroughly mixed in a 1.5 mL microcentrifuge tube with 20 µL of an *E. coli* sample. The mixture was incubated for 10 min at room temperature, and samples of the mixture in a 5 µL volume for each concentration were applied to microscopic glass slides. Agglutination was observed using a routine binocular microscope (Boeco model BM-120, Hamburg, Germany). A setup with objective magnification of 10× and lower illumination was used. Representative images were taken using a digital camera.

### 4.6. Biofilm Inhibition Assay

The biofilm inhibition potential of the phenolic compounds in the presence of Man was evaluated according to a previously described protocol [50], with slight modifications. The suspension of *E. coli* HB101(pPKL4) obtained as described in Section 4.2 was diluted in fresh LB medium at a 1:100 dilution. Then, 100 μL of the bacterial culture was added to each well of a 96-well microtiter plate and incubated for 4 h at 37 °C to allow cell attachment. Following incubation, 100 μL of 30 mM mannose or a mixture consisting of 30 mM mannose and a 0.25 mM solution of the phenolic compound (benzoic acid, chlorogenic acid, resveratrol, caffeic acid) was added to each well. An equal volume of phosphate-buffered saline (PBS) was added as a negative control, instead of the tested compounds. In blank wells, 200 μL of LB was used without a bacterial culture to ensure the sterility of the experiment. The plates were covered with a lid and incubated at 37 °C for 24 h. After incubation, the cultures were decanted onto a paper towel and rinsed two times with 200 μL sterile PBS at pH 7.2. Then, the plates were stained with 0.1% crystal violet (CV) solution for 20 min at room temperature. After this, the plates were washed three times with PBS at pH 7.2 to free the stain from the microtiter plates; they were then air-dried and destained with 200 μL of 95% ethanol (*v*/*v*) for about 30 min. Finally, the absorbance was measured at 590 nm with a Tecan Spark multimode microplate reader. The percentage inhibition of biofilm formation was calculated by using the following formula:

% inhibiton = (1 − OD_exp_/OD_neg. contr_) × 100.

## 5. Conclusions

Our study demonstrates the antiadhesive activity of cranberry-related phenolic compounds on FimH-mediated *E. coli* adhesion. Phenolic acids (benzoic, hippuric, *p*-coumaric, ferulic and caffeic), resveratrol, (+)-catechin, procyanidin A and a *V. macrocarpon* extract contributed to the inhibition of the agglutination of type 1 fimbriated *E. coli* and erythrocytes. Benzoic acid, chlorogenic acid, caffeic acid and resveratrol showed modest inhibitory potential regarding the formation of biofilms by FimH-mediated *E. coli*. Our further research efforts will be focused on (a) the use of more precise tests for the investigation of the influence of the corresponding phenolic compounds on FimH-mediated *E. coli* adhesion and (b) a structure–activity relationship study of mannose conjugates with benzoic acid, chlorogenic acid, caffeic acid, resveratrol and their derivatives with 3,4-dihydroxyphenyl motifs in the aglycone part. In the context of the rising prevalence and burden of resistant bacteria, identifying effective non-antibiotic strategies akin to this one is pivotal in reducing both colonization and infection risks, ultimately helping to preserve the efficacy of existing antimicrobial agents and mitigate the global AMR crisis.

## Figures and Tables

**Table 1:** Inhibition of the hemagglutination of guinea pig erythrocytes by type 1 fimbriated *E. coli* HB101(pPKL4) in the presence of mannose and 1 mM solutions of phenolic compounds.

Compound Tested	IT [mM]	Compound Tested	IT [mM]
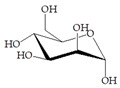 Man	60	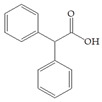 Man + diphenylacetic acid	60
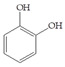 Man + catechol	60	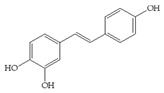 Man + resveratrol	30
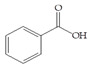 Man + benzoic acid	30	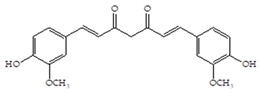 Man + curcumin	60
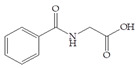 Man + hippuric acid	30	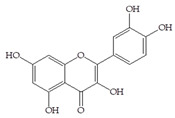 Man + quercetin	60
* 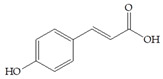 * Man + *p*-coumaric acid	30	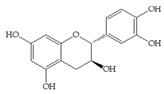 Man + (+)-catechin	30
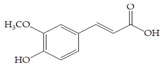 Man + ferulic acid	30	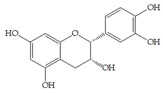 Man + (-)-epicatechin	60
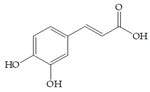 Man + caffeic acid	30	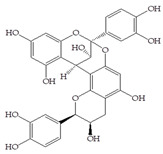 Man + procyanidin A2	30
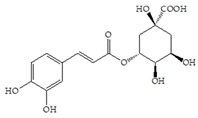 Man + chlorogenic acid	30	Man + *Vaccinium macrocarpon* extract (80 mg/mL)	30

**Table 2:** Inhibition of the hemagglutination of guinea pig erythrocytes by type 1 fimbriated *E. coli* HB101(pPKL4) in the presence of the most active phenolic compounds.

Compound Tested	IT [mM]
benzoic acid	0.25
hippuric acid	0.5
*p*-coumaric acid	0.5
ferulic acid	0.5
caffeic acid	0.25
chlorogenic acid	0.25
resveratrol	0.25
(+)-catechin	0.5
procyanidin A2	0.5
*Vaccinium macrocarpon* extract	10 ^1^

^1^ Expressed in g/mL.

## Data Availability

Dataset available on reasonable request from the authors.
